# Global metabolic alterations in colorectal cancer cells during irinotecan-induced DNA replication stress

**DOI:** 10.1186/s40170-022-00286-9

**Published:** 2022-07-04

**Authors:** Christian Marx, Jürgen Sonnemann, Oliver D. K. Maddocks, Lisa Marx-Blümel, Mandy Beyer, Doerte Hoelzer, René Thierbach, Claudia Maletzki, Michael Linnebacher, Thorsten Heinzel, Oliver H. Krämer

**Affiliations:** 1 Department of Toxicology, University Medical Center, Johannes Gutenberg University Mainz, Building 905, Mainz, Germany; 2 Department of Biochemistry, Center for Molecular Biomedicine (CMB), Institute for Biochemistry and Biophysics, Friedrich Schiller University of Jena, Jena, Germany; 3 Leibniz Institute on Aging - Fritz Lipmann Institute (FLI), Jena, Germany; 4 Current Address: Center for Pandemic Vaccines and Therapeutics (ZEPAI), Paul Ehrlich Institute, Langen, Germany; 5 Department of Paediatric Haematology and Oncology, Jena University Hospital, Children’s Clinic, Jena, Germany; 6 Research Center Lobeda, Jena University Hospital, Jena, Germany; 7 Wolfson Wohl Cancer Research Centre, Institute of Cancer Sciences, University of Glasgow, Glasgow, UK; 8 Department of Human Nutrition, Institute of Nutrition, Friedrich Schiller University of Jena, Jena, Germany; 9 Current address: Biopharmaceutical New Technologies (BioNTech) Corporation, Mainz, Germany; 10 Molecular Oncology and Immunotherapy, Thoracic, Vascular and Transplantation Surgery, Clinic of General, University of Rostock, VisceralRostock, Germany; 11 Current address: Department of Medicine, Clinic III - Hematology, Oncology, Palliative Medicine, Rostock University Medical Center, Rostock, Germany

**Keywords:** Adaptation, Colorectal cancer, Glucose, Irinotecan, Metabolism, p53, Warburg effect

## Abstract

**Background:**

Metabolic adaptations can allow cancer cells to survive DNA-damaging chemotherapy. This unmet clinical challenge is a potential vulnerability of cancer. Accordingly, there is an intense search for mechanisms that modulate cell metabolism during anti-tumor therapy. We set out to define how colorectal cancer CRC cells alter their metabolism upon DNA replication stress and whether this provides opportunities to eliminate such cells more efficiently.

**Methods:**

We incubated p53-positive and p53-negative permanent CRC cells and short-term cultured primary CRC cells with the topoisomerase-1 inhibitor irinotecan and other drugs that cause DNA replication stress and consequently DNA damage. We analyzed pro-apoptotic mitochondrial membrane depolarization and cell death with flow cytometry. We evaluated cellular metabolism with immunoblotting of electron transport chain (ETC) complex subunits, analysis of mitochondrial mRNA expression by qPCR, MTT assay, measurements of oxygen consumption and reactive oxygen species (ROS), and metabolic flux analysis with the Seahorse platform. Global metabolic alterations were assessed using targeted mass spectrometric analysis of extra- and intracellular metabolites.

**Results:**

Chemotherapeutics that cause DNA replication stress induce metabolic changes in p53-positive and p53-negative CRC cells. Irinotecan enhances glycolysis, oxygen consumption, mitochondrial ETC activation, and ROS production in CRC cells. This is connected to increased levels of electron transport chain complexes involving mitochondrial translation. Mass spectrometric analysis reveals global metabolic adaptations of CRC cells to irinotecan, including the glycolysis, tricarboxylic acid cycle, and pentose phosphate pathways. P53-proficient CRC cells, however, have a more active metabolism upon DNA replication stress than their p53-deficient counterparts. This metabolic switch is a vulnerability of p53-positive cells to irinotecan-induced apoptosis under glucose-restricted conditions.

**Conclusion:**

Drugs that cause DNA replication stress increase the metabolism of CRC cells. Glucose restriction might improve the effectiveness of classical chemotherapy against p53-positive CRC cells.

**Graphical Abstract:**

The topoisomerase-1 inhibitor irinotecan and other chemotherapeutics that cause DNA damage induce metabolic adaptations in colorectal cancer (CRC) cells irrespective of their p53 status. Irinotecan enhances the glycolysis and oxygen consumption in CRC cells to deliver energy and biomolecules necessary for DNA repair and their survival. Compared to p53-deficient cells, p53-proficient CRC cells have a more active metabolism and use their intracellular metabolites more extensively. This metabolic switch creates a vulnerability to chemotherapy under glucose-restricted conditions for p53-positive cells. 
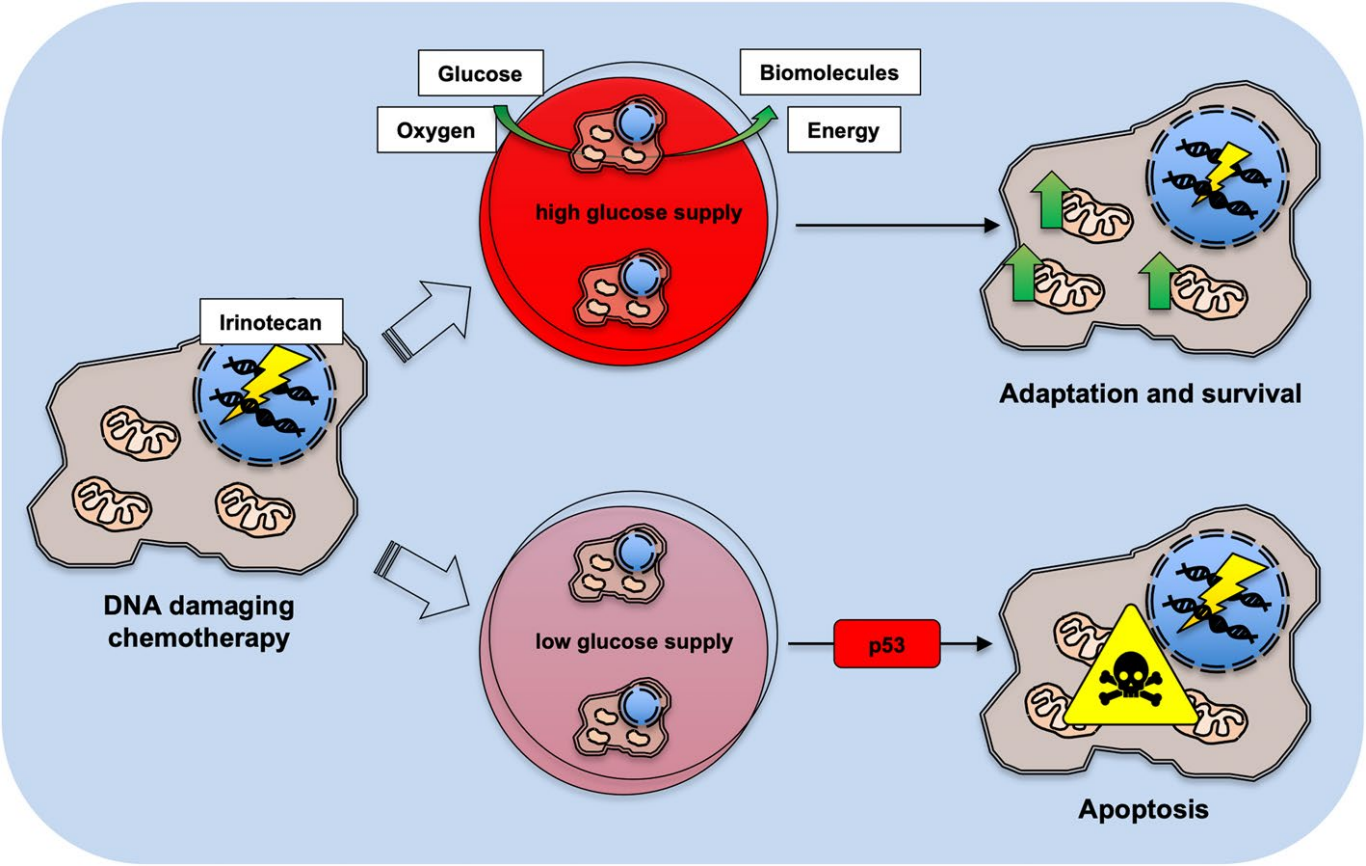

**Supplementary Information:**

The online version contains supplementary material available at 10.1186/s40170-022-00286-9.

## Introduction

Metabolism serves to fuel cells with energy and the building blocks for biosynthesis. The fine-tuned balance between catabolic and anabolic processes involves several interconnected pathways, with mitochondria as cellular powerhouses for efficient energy retrieval. Glucose, a main source of cellular energy, is degraded via glycolysis or metabolized within the pentose phosphate pathway (PPP) [1, 2]. Glycolysis produces two molecules of pyruvate and of the energy-rich molecules ATP and NADH. This process consumes two molecules ADP and NAD^+^ per molecule glucose. To quickly regenerate NAD^+^, cells can metabolize pyruvate to lactate [1]. The more efficient, but slower oxidative turnover of pyruvate fuels the tricarboxylic acid (TCA) cycle and the oxidative phosphorylation (OXPHOS) within the mitochondrial electron transport chain (ETC). This pathway yields about 32 molecules of ATP per molecule glucose [1, 3]. ETC enzymes, like complex IV, are regulated allosterically to adjust the cell metabolism to the cellular energy level and to the availability of metabolites [4].

Despite a higher ATP yield from mitochondrial respiration over glycolysis, cancer cells tend to perform glycolysis even in the presence of sufficient oxygen. In the 1920s, Otto Warburg first described this hallmark of cancer, which is called aerobic glycolysis or the Warburg effect [1, 5–7]. This glycolytic metabolism delivers ATP and molecules for biosynthesis faster without reactive oxygen species (ROS) production, which can cause lethal DNA damage in fast dividing cancer cells that frequently have mutations in DNA repair proteins [6, 8–10]. In addition, this dysregulated cell metabolism supports fast cancer cell proliferation, even under hypoxic conditions in solid tumor masses [1, 10–12]. Nevertheless, cancer cells retain a certain capacity to activate their OXPHOS after stress, e.g., for DNA repair, and cancer cell metabolism is more complex and flexible than initially believed [1, 6, 7, 10, 13–17]. Hence, metabolic adaptations can help cancer cells to escape DNA-damaging drugs [3, 10, 18–22]. Damages to the ETC and mitochondrial DNA (mtDNA) can in turn increase the chemosensitivity of tumor cells [3, 10]. Accordingly, there is an intense search for mechanisms and metabolic inhibitors for anti-tumor therapy [16, 23].

Colorectal cancer (CRC) is the second most common cause of cancer death. Alone in the USA, approximately 147,950 individuals were diagnosed with CRC and 53,200 died from the disease in 2020 [24]. Defining and exploiting metabolic adaptation might save millions of lives each year. It is reported that caloric restriction (CR) and fasting periods increase the effectiveness of chemotherapy, by protecting non-malignant cells and by increasing autophagy and apoptosis in tumor cells [25–27]. However, it is still a matter of debate whether CR or fasting in combination with chemotherapy may have a beneficial or an unfavorable outcome [28].

The tumor suppressor protein p53 is a master regulator of cell metabolism, mitochondrial integrity, and antioxidant responses to ROS. Moreover, p53 regulates key metabolic pathways including the OXPHOS, glycolysis, PPP, TCA cycle, and glutaminolysis under physiologic conditions and during stress [8–10, 29–31].Whether p53 affects metabolic adaptation of CRC cells upon chemotherapy with drugs that cause DNA replication stress is unclear.

We set out to investigate metabolic alterations after drug-induced DNA replication stress, and the role of p53 in these processes. We analyzed the responses of human p53 wild-type (HCT116^wt^) and otherwise isogenic p53 null (HCT116^Δp53^) HCT116 CRC cells to clinically approved DNA-damaging chemotherapeutics. These include the topoisomerase-1 (TOP1) inhibitor irinotecan, the ribonucleotide reductase (RNR) inhibitor hydroxyurea, and the topoisomerase-2 (TOP2) inhibitor doxorubicin [32–35]. Independent of p53, CRC cells increased their metabolic activities when exposed to these agents. Compared to p53-negative cells, p53-proficient cells were more reliant on extracellular glucose level for their survival upon DNA replication stress.

## Results

### Irinotecan treatment leads to increased mitochondrial activity

**Fig. 1:**
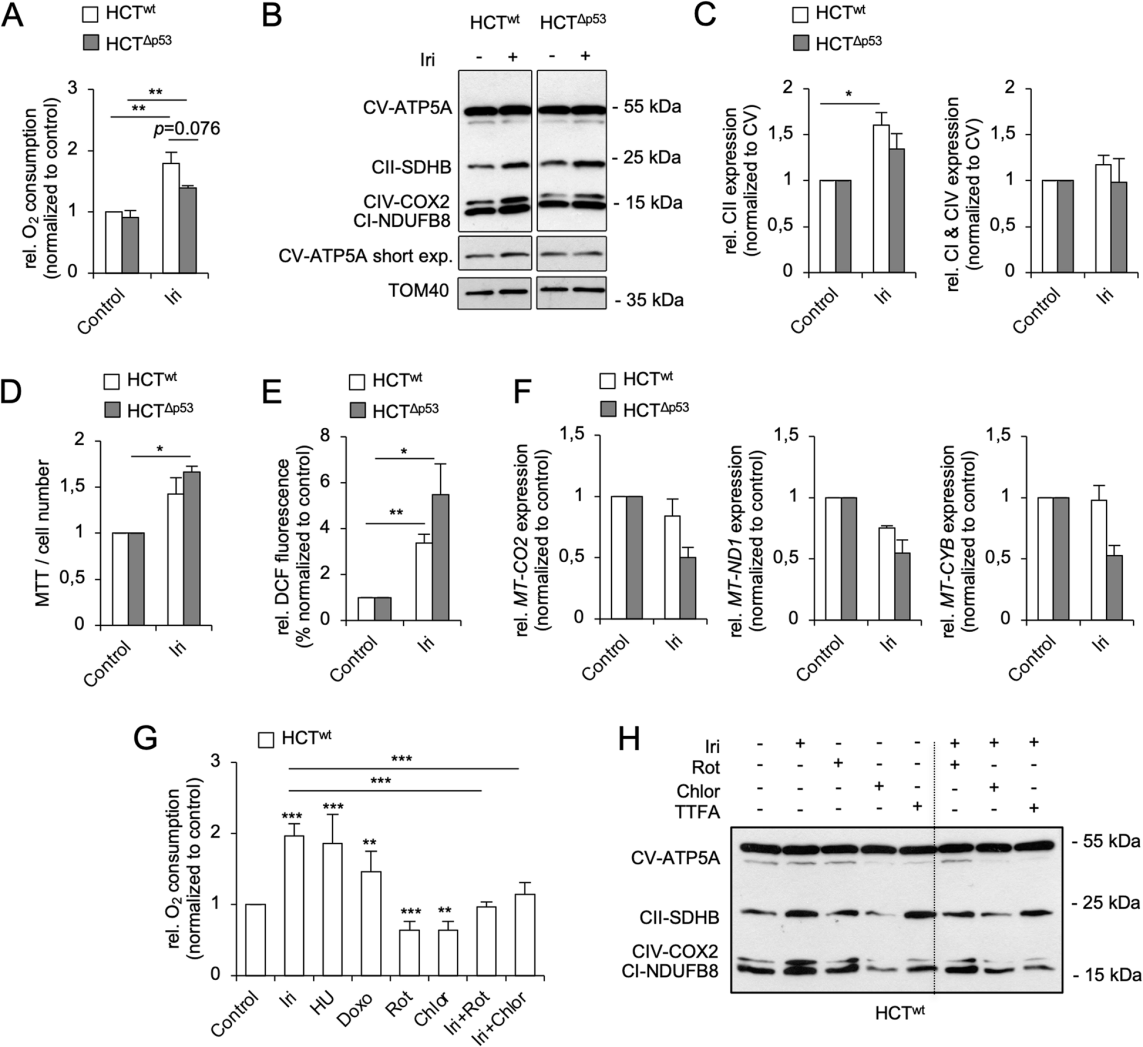
DNA-damaging chemotherapeutics increase the metabolism of HCT116 cells. **A**–**F** p53 wild-type (HCT^wt^) and isogenic p53 null (HCT^Δp53^) HCT116 cells were exposed to 10 µM irinotecan (Iri) for 24 h. **A** The oxygen consumption of cells was assessed using a Clarke electrode. **B** Cell fractionations were prepared and the expression of indicated proteins in equal amounts of mitochondrial extracts was analyzed by Western blot. TOM40 was used to control mitochondrial protein loading. The relative expression of electron transport chain (ETC) complex subunits to complex V was quantified by densitometry using ImageJ and is depicted in **C**. **D** MTT assays were performed, and corresponding cell numbers counted in parallel (refer to Fig. S1D) to calculate the MTT turnover/cell number. **E** The accumulation of reactive oxygen species (ROS) in cells was determined using flow cytometry. **F** The mRNA expression of indicated genes was assessed by qPCR. **G**, **H** HCT^wt^ cells were exposed to 10 µM Iri, 1 µM hydroxyurea (HU), 1 µM doxorubicin (Doxo), 0.1 mM TTFA, 0.1 µM rotenone (Rot), or 0.1 mg/ml chloramphenicol (Chlor) as single agents and in combination for 24 h. **G** The oxygen consumption of cells was assessed using a Clarke electrode. **H** The expression of indicated proteins within mitochondrial extracts was analyzed by Western blot. **A** The average of 4 individual experiments ± SEM. **C**–**E**, **G** The average of 3 individual experiments ± SEM. **F**, **H** The average of /is representative for 2 individual experiments ± SEM. Statistics for this figure: *p < 0.05; **p < 0.01; ***p < 0.001

Metabolic processes can determine the fate of tumor cells with DNA replication stress, but it is ill defined how irinotecan modulates such adaptations. Therefore, we asked if irinotecan alters mitochondrial functions and used HCT116^wt^ and HCT116^Δp53^ CRC cells as testbed. A direct way to measure the metabolism and ETC activity is to analyze mitochondrial oxygen consumption in cells. We found that irinotecan significantly increased this parameter after 24-h treatment in both HCT116 cell lines; HCT116^wt^ cells showed a trend to higher respiration than HCT116^Δp53^ cells (Fig. 1A). This was not due to differences in the accumulation of DNA replication stress/DNA damage, as evidenced by a similar accumulation of ɣH2AX in irinotecan-treated CRC cells with or without p53 (Fig. S1A). Moreover, oxygen consumption did not increase during the first 4 h after the application of irinotecan (Fig. S1B), indicating an adaptive process over time.

Oxygen is consumed in the ETC complex IV and requires an electron flow through the first four ETC complexes [1, 3, 36, 37]. To study whether irinotecan altered the protein levels of ETC complex subunits, we analyzed mitochondrial extracts by immunoblotting and compared ETC protein expressions to the mitochondrial outer membrane protein TOM40 (Fig. 1B, C). While ETC complex V (CV; ATP synthase subunit ATP5A) expression was not affected by irinotecan, the expression levels of complex I (CI; NADH dehydrogenase (NDH) subunit NDUFB8), II (CII; succinate dehydrogenase (SDH) subunit SDHB), and IV (CIV; cytochrome *c* oxidase (COX) subunit 2) subunits were increased relative to complex V in both HCT116 cell lines upon treatment with irinotecan. ETC CII, which is part of both the TCA cycle and the OXHPOS machinery [1, 3, 36, 37], showed the strongest increase after irinotecan treatment (Fig. 1B, C). As seen for oxygen consumption rates (Fig. 1A), HCT116^wt^ cells showed a higher increase in ETC protein expression than p53-deficient cells in response to irinotecan.

To see whether this induction of ETC complexes also occurs in vivo, we transplanted short-term cultured human CRC cells (HROC24 (p53 wild-type) and HROC87 (p53 mutated) [38]) in mice, treated them with irinotecan, excised the tumors, and probed for ETC proteins by immunoblot. Like in cultured cells, irinotecan augmented the expression of ETC proteins in xenografted CRC cells (Fig. S1C).

Figure 1B illustrates that irinotecan particularly induces the expression of ETC CII in HCT116 cells. For a functional analysis, we studied the regulation of the ETC CII by irinotecan with the MTT assay, which also gives information about the intracellular redox balance [39]. In parallel, cell numbers were counted to subtract proliferation-dependent effects. We observed an increased MTT/cell number turnover, which was higher in HCT116^Δp53^ cells (Figs. 1D and S1D). Since HCT116^wt^ cells showed higher ETC protein expressions and OXPHOS activities than p53-deficient cells (Fig. 1A), we tested if the high MTT/cell number turnover originated from changes of the intracellular redox balance. The measurement of cytosolic ROS level by flow cytometry indeed showed that irinotecan induced a significant ROS increase in both CRC cell lines, with HCT116^Δp53^ cells being more affected (Fig. 1E).

These data indicate that irinotecan induces an increase in ETC activity in CRC, which is accompanied by an accumulation of ROS, and that p53 fine-tunes these effects.

### Irinotecan treatment impairs mitochondrial gene transcription

Mitochondria are the primary source and a target of ROS-induced damages [40, 41]. If the increased ETC protein levels after irinotecan treatment (Fig. 1B, C) are due to an increased transcription of mtDNA-encoded genes or an increase in mitochondria *per se*, there should be an increased number of mitochondrial mRNAs after irinotecan treatment. The human mtDNA harbors 39 genes, of which 13 code for the essential core subunits of the ETC complexes: I (*MT-ND1*, *MT-ND2*, *MT-ND3*, *MT-ND4*, *MT-ND4L*, *MT-ND5*, and *MT-ND6*), III (*MT-CYB*), IV (*MT-COI*, *MT-COII*, and *MT-COIII*), and V (*MT-ATP6* and *MT-ATP8*) (MITOMAP: a human mitochondrial genome database; http://www.mitomap.org). The assembly of ETC complexes is a highly complicated and well-coordinated process. Changes in the expression of the mtDNA-encoded subunits affect the assembly of the whole ETC super-complexes [36, 37].

Analyses with quantitative polymerase chain reaction (qPCR) showed that irinotecan decreased the mitochondrial encoded *MT-COII* (COX2), *MT-ND1* (NDH subunit 1), and *MT-CYB* (cytochrome *b*) mRNA levels in HCT116^Δp53^ cells (Fig. 1F). HCT116^wt^ cells had moderately decreased *MT-COII* and *MT-ND1* expression levels after irinotecan treatment, disfavoring that irinotecan enhanced the mtDNA gene expression.

This decrease of mtDNA transcripts (Fig. 1F) made us hypothesize that increased translation contributed to the higher levels of ETC proteins and increased respiration. We used chloramphenicol, which inhibits mitochondrial ribosomes [42], to analyze whether irinotecan increases the mitochondrial protein translation (Figs. 1G, H, and S1E). Congruent with our hypothesis, chloramphenicol reduced the oxygen consumption in both irinotecan-treated HCT116 cell lines significantly, and chloramphenicol alone as well as in combination with irinotecan suppressed the expression of ETC CI-IV. The expression of ETC CV was again unaffected by any treatment (Fig. 1H), indicating a longer protein half-life compared with the other tested ETC complex subunits. In addition, the use of the ETC CI inhibitor rotenone and the ETC CII inhibitor 2-thenoyltrifluoroacetone (TTFA) [43] showed that the activity of both ETC complexes significantly contributed to the DNA damage-induced increase in oxygen consumption (Figs. 1G and S1E-F). Noteworthy, neither rotenone nor TTFA suppressed the induction of ETC CI-IV proteins by irinotecan (Fig. 1H).

We also measured the mitochondrial respiration after exposure to hydroxyurea and doxorubicin, which cause DNA replication stress and DNA damage [33, 34]. Both agents increased the oxygen consumption significantly and p53-independently (Figs. 1G and S1E), indicating a general increase of respiration after DNA replication stress/DNA damage.

These data illustrate that enhanced ETC protein expression in response to irinotecan is connected to the translation of mtDNA.

### Irinotecan increases both glycolysis and respiration in HCT116 cells

We speculated that irinotecan might induce global metabolic adaptations. To test this, we analyzed the metabolic flux of HCT116 cells in real time with a Seahorse Analyzer. This system allows parallel measuring of oxygen consumption (OCR) and extracellular acidification rates (ECAR). The latter results from fermentative glycolysis and lactate secretion [44, 45].

**Fig. 2:**
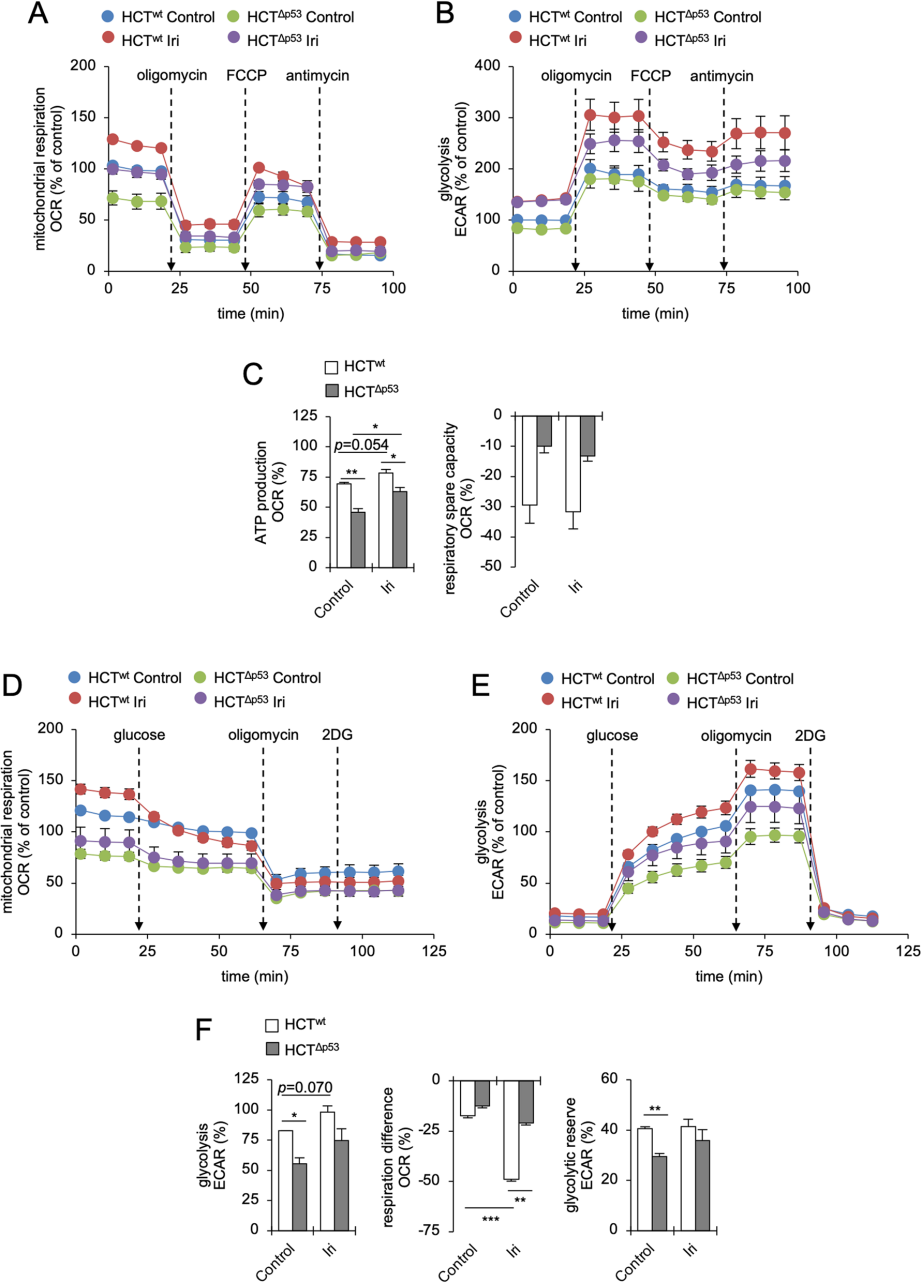
Irinotecan activates both respiration and glycolysis in HCT116 cells. p53 wild-type (HCT^wt^) and isogenic p53 null (HCT^Δp53^) HCT116 cells were exposed to 10 µM irinotecan (Iri) for 24 h. **A** Oxygen consumption (OCR) and **B** extracellular acidification rates (ECAR) were assessed with a Cell Mito Stress Test using a Seahorse XFe24 Analyzer. 2 µM oligomycin, 2 µM FCCP, and 2 µM antimycin A were injected consecutively during the measurement. **C** Bioenergetic parameters, i.e., ATP production and respiratory spare capacity, were calculated from OCR/ECAR curves shown in **A** and **B**. **D** OCR and **E** ECAR were assessed with a Glycolysis Stress Test using a Seahorse XFe24 Analyzer. 10 mM d-glucose, 2 µM oligomycin, and 50 mM 2-DG were injected consecutively during the measurement. **F** Bioenergetic parameters, i.e., glycolysis, respiration difference (before and after glucose injection) and glycolytic reserve, were calculated from OCR/ECAR curves shown in **D** and **E**. All figures show the average of 3 individual experiments ± SEM. Statistics for this figure: *p < 0.05; **p < 0.01; ***p < 0.001

To study mitochondrial parameters, we performed a Cell Mito Stress Test (Figs. 2A–C and S2A-B). We found that HCT116^wt^ cells had higher basal OCR and ECAR than p53-deficient cells (Figs. 2A, B, and S2B). Irinotecan increased these values in both CRC cell lines significantly (Figs. 2A, B, and S2B). The ATP production in HCT116^wt^ cells was higher than in cells without p53, and irinotecan increased this parameter significantly in both CRC cell lines (Fig. 2C). Unexpectedly, the respiratory spare capacity was negative in both HCT116 cell lines and lower in p53 wild-type cells (Fig. 2A, C). This suggests that HCT116 cells preferentially use glycolysis and cannot sustain their mitochondrial integrity upon DNA replication stress. Further details on this aspect are provided below.

Irinotecan also increased the metabolism of p53 wild-type RKO CRC cells (Fig. S2C-D). Unlike HCT116 cells, these cells mainly increased their OXPHOS activity in response to the DNA replication stress, which was accompanied by an increased ATP production and respiratory spare capacity (Fig. S2E).

To study glycolytic functions in more detail, we performed a Glycolysis Stress Test (Figs. 2D–F and S2F-G). We found that during the first period of this assay, when l-glutamine was the only available energy resource, both irinotecan-treated and control HCT116^wt^ cells had a significantly higher OCR (glutamine-driven respiration) than HCT116^Δp53^ cells (Figs. 2D–F and S2F-G). After the addition of glucose, HCT116^wt^ cells maintained a higher OCR (glucose-driven respiration) and ECAR (glycolysis) than p53-deficient cells (Figs. 2D–F and S2F-G). Although irinotecan enhanced ECAR values in both HCT116 cell lines, we observed a significant decrease of OCR specifically in irinotecan-treated HCT116^wt^ cells (50.2% OCR decrease) after the addition of glucose (Figs. 2D, F, and S2G). HCT116^Δp53^ cells did not show this response to glucose (Fig. 2D). The glycolytic reserve, which indicates the capacity of cells to compensate OXPHOS defects by glycolysis, was significantly higher in untreated p53-proficient cells than in HCT116^Δp53^ cells (Fig. 2F).

Finally, to separate and analyze mitochondrial functions of HCT116 cells under conditions supporting either a glycolysis- or an OXPHOS-driven metabolism, we customized a Cell Mito Stress Test. On the one hand, cells were supplied with glucose (no pyruvate in the medium) feeding the (aerobic) glycolysis and the TCA cycle. On the other hand, cells were supplied with pyruvate (no glucose in the medium) selectively feeding the TCA cycle and subsequently the OXPHOS. Both conditions were analyzed side-by-side in control and irinotecan-treated HCT116 cells (Fig. S3). In the first setting, when glucose was the main energy source (Fig. S3A–E), we made similar observations as seen before (compare with Figs. 2A–C and S2A-B): p53-proficient cells had a more active metabolism than p53-deficient cells, both had negative respiratory spare capacities, and the treatment with irinotecan increased the OCR of HCT116 cells (Fig. S3B-E). Intriguingly, we found in the second setting (Fig. S3F-J) that HCT116 cells had a significantly higher OCR than before (about 100% increase), which was further increased especially in irinotecan-treated HCT116^wt^ cells. In addition, the mitochondrial ATP production was greatly increased, and cells shifted into prominent and positive respiratory spare capacities (compare Figs. 2A–C, S3B, and E with G and J). The treatment with irinotecan increased the ATP production and respiratory spare capacities particularly in p53-proficient HCT116 cells. As expected, ECAR values were dramatically decreased in the absence of glucose confirming that only small amounts of pyruvate were converted into lactate and mainly used to feed the TCA cycle and OXPHOS (Fig. S3H).

These data show that despite high levels of respiration, p53-positive CRC cells switch to glycolysis in the presence of glucose. During DNA replication stress, HCT116^wt^ cells prefer to consume glucose aerobically and are less reliant on their mitochondria. If glucose is not available, these cells strongly increase their respiration.

### Mass spectrometry reveals global metabolic adaptations to DNA replication stress/DNA damage

**Fig. 3:**
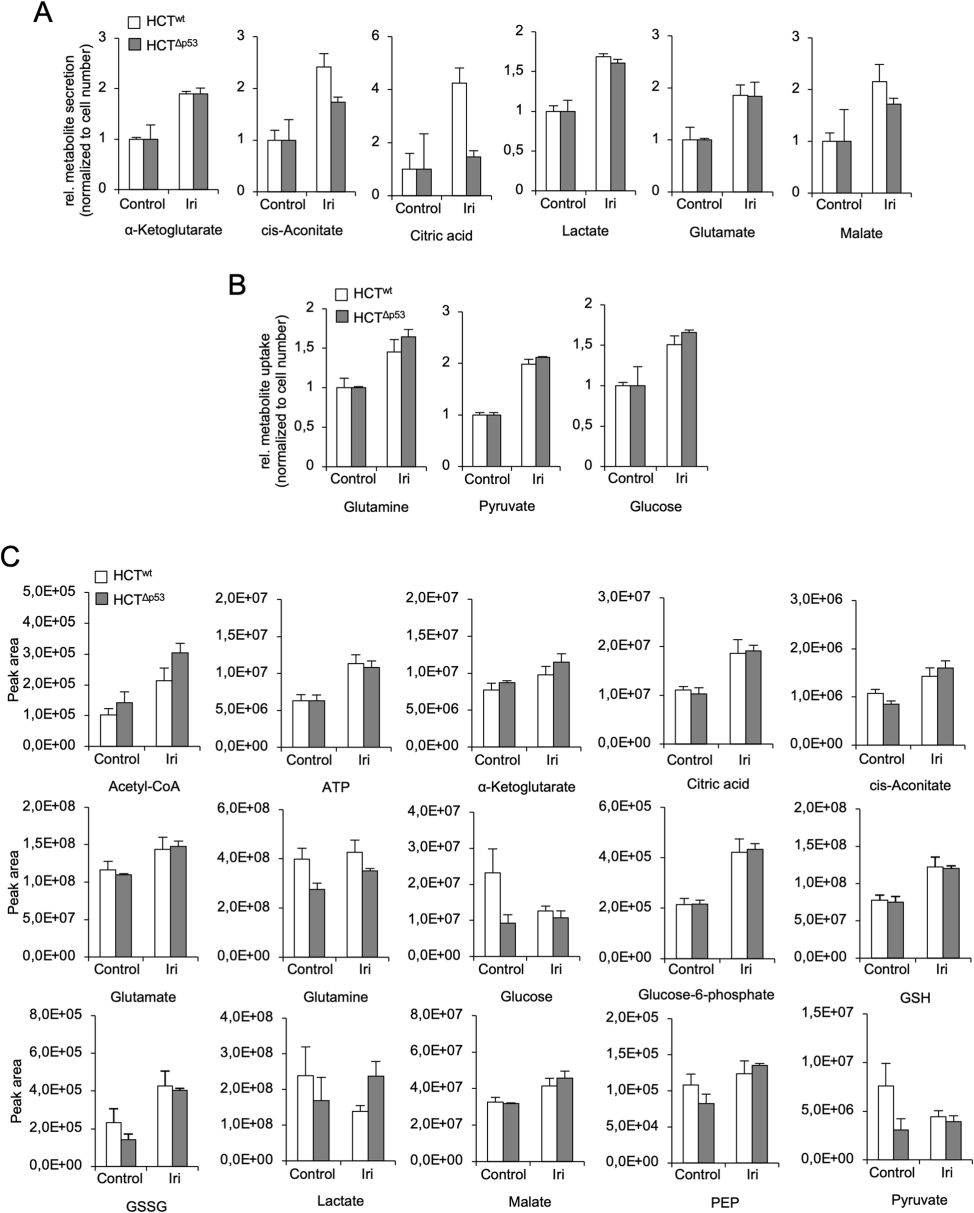
Mass spectrometry confirms increased cell metabolism after irinotecan treatment. **A**–**C** p53 wild-type (HCT^wt^) and isogenic p53 null (HCT^Δp53^) HCT116 cells were exposed to 10 µM irinotecan (Iri). The abundance of indicated metabolites in the supernatant cell culture medium (**A**, **B**) and in cells (**C**) was assessed after 24 h by targeted mass spectrometric analysis (metabolomics). Alterations of metabolite secretion and uptake after treatment are depicted in **A** and **B**, respectively. Metabolites present in the DMEM medium (high glucose) at the beginning of the experiment were subtracted from the date shown in **A** and **B**. Changes of intracellular metabolites are shown in **C**. Abbreviations: GSH/GSSG reduced/oxidized glutathione, PEP phosphoenolpyruvate. **A**–**C** The average of 3 individual experiments ± SEM

**Fig. 4:**
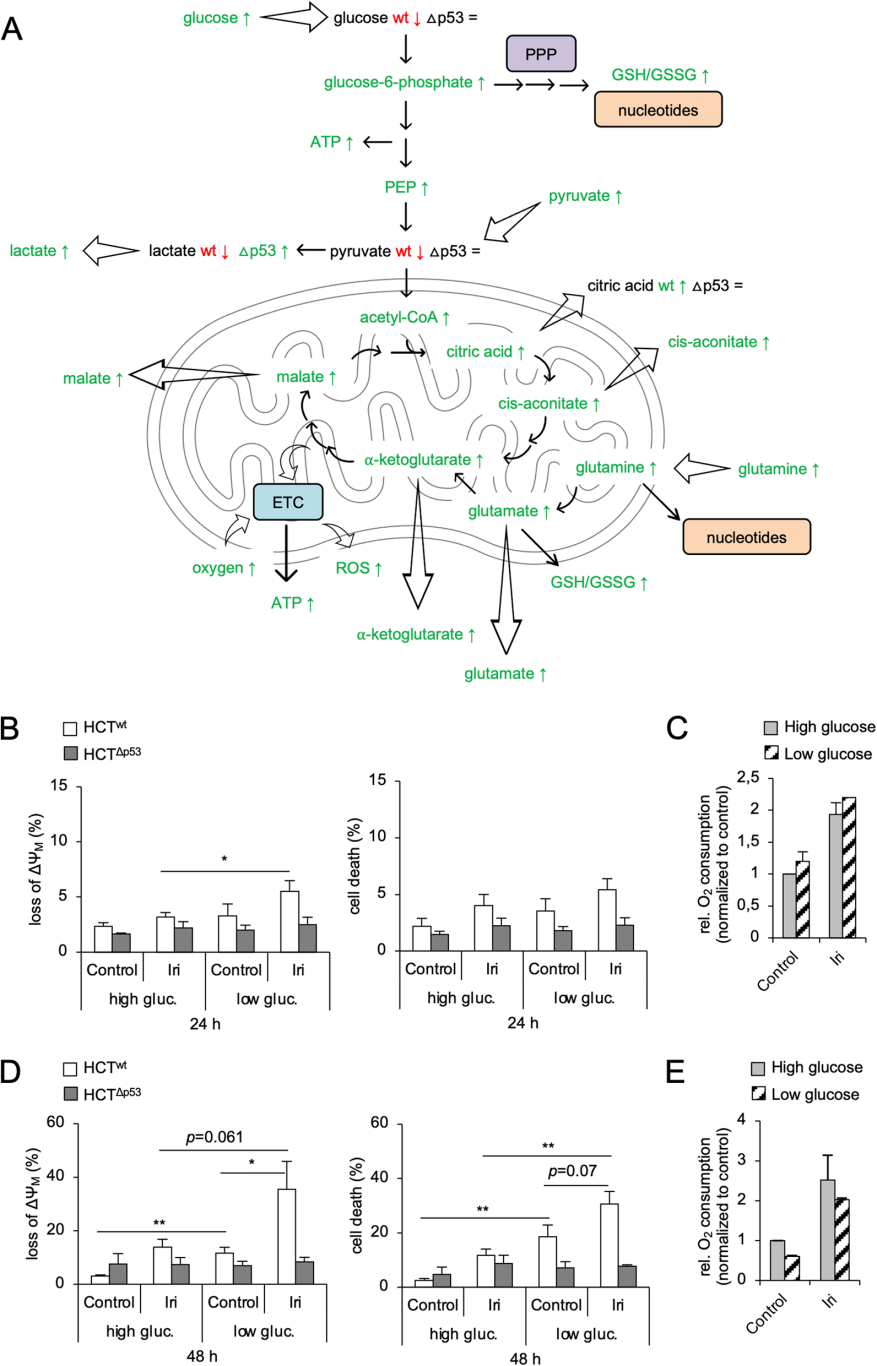
Glucose restriction sensitizes p53-proficient HCT116 cells to irinotecan. **A** p53 wild-type (HCT^wt^) and isogenic p53 null (HCT^Δp53^) HCT116 cells were exposed to 10 µM irinotecan (Iri). The abundance of indicated metabolites was assessed after 24 h by targeted mass spectrometric analysis (metabolomics) (refer to Fig. 3). Changes of individual metabolites are summarized for the HCT116 cell lines. Green/red color indicates a higher/lower abundance after irinotecan treatment, respectively. Black indicates no change after treatment. If not further specified, metabolites in both HCT^wt^ (wt) and HCT^Δp53^ (Δp53) were altered in the same way. Abbreviations: ETC electron transport chain, PPP pentose phosphate pathway, ROS reactive oxygen species, GSH/GSSG reduced/oxidized glutathione, PEP phosphoenolpyruvate. **C**–**E** HCT^wt^ and isogenic HCT^Δp53^ cells cultured in DMEM with high (4.5 g/l) or low (1 g/l) glucose were exposed to 10 µM Iri. The loss of mitochondrial membrane potential (loss of ΔΨ_M_) and cell death was analyzed by flow cytometry after 24 h (**B**) and 48 h (**D**). The oxygen consumption of HCT^wt^ cells was assessed after 24 h (**C**) and 48 h (**E**) using a Clarke electrode. **B**, **D** The average of 4 individual experiments ± SEM. **C**, **E** The average of 2 individual experiments ± SEM. Statistics for this figure: *p < 0.05; **p < 0.01

To study the cell metabolism of irinotecan-treated HCT116 cells in a global manner, we performed a targeted mass spectrometric analysis of selected metabolites (metabolomics). After 24 h, we analyzed changes of metabolites in the cell culture medium (Fig. 3A, B) as well as in irinotecan-treated HCT116 cells (Fig. 3C). The results of our analysis are summarized in Fig. 4A.

Both CRC cell lines consumed higher levels of glutamine, pyruvate, and glucose and secreted more α-ketoglutarate, cis-aconitate, citric acid, lactate, glutamate, and malate from/into the culture medium after irinotecan application. HCT116^wt^ cells secreted more cis-aconitate, citric acid, and malate than HCT116^Δp53^ cells (Fig. 3A, B). Among intracellular metabolites, we observed that acetyl-CoA, α-ketoglutarate, cis-aconitate, citric acid, glutamine/glutamate, glucose-6-phosphate, malate, and phosphoenolpyruvate (PEP) increased in both HCT116 cell lines similarly after irinotecan treatment together with intracellular ATP level (Fig. 3C). This data is congruent to our Seahorse analysis (Fig. 2). High amounts of glucose-6-phosphate together with an increased level of reduced and oxidized glutathione (GSH/GSSG) show that irinotecan also activated the PPP (Fig. 3C).

Intriguingly, we found that untreated HCT116^wt^ cells had a higher level of intracellular glucose, pyruvate, and lactate than p53-deficient cells. All these metabolites decreased in HCT116^wt^ cells after irinotecan treatment (Fig. 3C). In contrast to this, glucose and pyruvate levels in HCT116^Δp53^ cells were stable after irinotecan application, whereas lactate level moderately increased.

These results illustrate that the irinotecan-induced DNA replication stress globally activates several metabolic pathways and that p53-proficient CRC cells use their metabolites in a more extensive manner.

### HCT116^wt^ cells rely on glucose for their survival after DNA damage

The data above show that p53-proficient HCT116 cells have a significantly higher (glycolytic) metabolism after DNA replication stress/DNA damage than HCT116^Δp53^ cells (Figs. 1, 2, 3, and 4A). Thus, we investigated if HCT116^wt^ cells could be sensitized to irinotecan by lowering glucose availability. We cultivated both HCT116 cell lines in medium with high (4.5 g/l) or low (1 g/l) glucose and treated them for up to 48 h with irinotecan (Fig. 4B–E). We measured mitochondrial depolarization (loss of ΔΨ_M_) as an early apoptosis marker and PI uptake for total cell death by flow cytometry. After 24-h treatment with irinotecan in a low-glucose medium, HCT116^wt^ cells showed significantly higher levels of ΔΨ_M_ loss than cells that were cultivated in high glucose (Fig. 4B). The oxygen consumption of these cells was slightly increased when cultured in a medium with low glucose (Fig. 4C). Compared to cells cultivated in high glucose, irinotecan-treated HCT116^wt^ cells in a medium with low glucose had significantly higher levels of ΔΨ_M_ loss and cell death after 48 h (Fig. 4D). In addition, we observed that untreated HCT116^wt^ cells in the low-glucose medium had significantly more ΔΨ_M_ loss and cell death than cells that were cultured in the high-glucose medium. This correlates with a decreased oxygen consumption of HCT116^wt^ cells after 48 h of culture in a medium with low glucose levels (Fig. 4E) suggesting that the remaining glucose level in the culture medium of p53-positive cells after 48 h was insufficient to fuel their mitochondrial metabolism properly and might become a limiting factor for these cells. HCT116^Δp53^ cells did not show any significant increase in cell death or ΔΨ_M_ loss after 24–48-h irinotecan treatment, irrespective of glucose levels in the culture media (Fig. 4B, D). These data show that p53-proficient CRC cells need glucose to remain viable during DNA replication stress/DNA damage.

## Discussion

Irinotecan is frequently applied to treat CRC. Unfortunately, it produces severe side effects that compromise its use [32, 35, 46]. Fasting can reduce the severity of such side effects without affecting its anti-tumor efficiency in xenograft mouse models [47]. Moreover, CR and fasting were shown to increase the anti-tumor effectiveness of chemotherapy [25–27]. Thus, the tumor metabolism is an appealing target in combination with classical chemotherapeutic agents to improve the treatment outcome in patients [14–16, 20, 26, 28, 48].

The common view on cancer cell metabolism is that malignant cells increase their glycolysis and decrease their respiration during transformation to reduce intracellular ROS level and to proliferate quickly, which is one hallmark of cancer [1, 5, 6, 11, 13]. Nevertheless, cancer cell metabolism is more complex and flexible [1–3, 6, 10, 13, 16–18, 30, 49]. In this context, we asked how irinotecan changes mitochondrial functions of CRC cells and how we could modulate its effectiveness via manipulating their metabolism. We show that the clinically approved drugs [32–34], irinotecan, hydroxyurea, and doxorubicin, increase the respiration of CRC cells irrespective of their p53 status. Furthermore, we demonstrate that DNA replication stress/DNA damage activates global metabolic alterations in CRC cells that help them to deal with the genome injury. These data are congruent with the finding that DDR signaling can activate SIRT1/AMPK/PGC1α-mediated molecular pathways to increase mitochondrial functions and biogenesis [50–52]. Similar with our data, others found with the MTT assay that a direct induction of DNA breaks by ɣ-irradiation augmented ETC CII activity and mitochondrial biogenesis in cervix and breast cancer cells [53]. According to our data, irinotecan increases the ETC activity and causes an accumulation of ETC complexes that requires apparently increased translation of reduced amounts of mitochondrial mRNAs. These data suggest that irinotecan promotes the ETC activity differently from ɣ-irradiation-induced DNA damages and likely independent of mitochondrial biogenesis. Further studies are necessary to reveal if direct DNA damage (ɣ-irradiation) changes mitochondrial functions differently compared to DNA replication stress and indirect DNA damage (irinotecan). Moreover, cell type-specific differences may determine how mitochondria respond to DNA replication stress and DNA damage.

Our Seahorse analysis unraveled that HCT116 cells increased both their glycolysis and respiration during DNA replication stress. HCT116^wt^ cells had in general a higher metabolism than p53-deficient cells and preferentially increased their glycolysis during DNA replication stress when glucose was available. This is in line with a recent study in hepatocellular carcinomas (HCCs) [31] but in contrast to the classical view on p53-mediated metabolic changes in response to stress [8, 9, 29, 30]. The difference may originate from the KRAS^G13D^ mutation in HCT116 cells. CRC tumors and cell lines have frequently mutations in *KRAS* leading to metabolic reprogramming, including an increased glycolysis, PPP activity, and glutaminolysis, making them reliant on these pathways [10, 41, 54–56]. In comparison, p53 and KRAS wild-type RKO cells [57] just increased their respiration due to irinotecan-induced DNA replication stress, without prominent effects on their glycolysis. The metabolic rewiring due to *KRAS* mutations in CRC translates into differential activation of molecular signaling pathways like mTOR and AMPK and causes metabolic maladaptation in patients [10, 41, 54, 55]. This should be considered for personalized treatment approaches that target the tumor metabolism in parallel to classical chemotherapy.

Using a customized Seahorse analysis, we demonstrate that HCT116 cells do not reconstitute their OXPHOS activity after mitochondrial uncoupling via cyanide-4-(trifluoromethoxy)-phenylhydrazone (FCCP) injection when glucose is available. Nevertheless, when cells are glucose-restricted, they use pyruvate to selectively fuel their TCA cycle and OXPHOS. Thus, they have a greater basal OCR than before and show a strong respiratory spare capacity after mitochondrial uncoupling. This indicates that HCT116 cells generate their energy solely from glycolysis when their mitochondrial ATP synthase is inhibited by the injection of oligomycin. In the Mito Stress Test, they do not rescue their mitochondrial membrane potential by increasing their OXPHOS after mitochondrial uncoupling. This highlights the addiction of HCT116 cells to glucose as their key energy source, being a textbook example of a colorectal cancer showing the Warburg effect [1, 5–7]. When glucose is not available, these cells are forced to utilize their mitochondrial potential more extensively.

We further reveal that the irinotecan-induced mitochondrial activity led to high ROS levels. Regarding ROS, p53 is a well-known regulator of cell metabolism and the antioxidant response [8–10, 29]. Confirming this notion, less ROS accumulated in HCT116^wt^ cells after irinotecan treatment compared with their p53-deficient counterparts, although HCT116^wt^ showed a stronger OXPHOS increase. This tied in with a more pronounced reduction of *MT-COII*, *MT-ND1*, and *MT-CYB* in irinotecan-treated HCT116 cells lacking p53. ROS originate mainly from ETC CI and CIII during OXPHOS and damage the mtDNA directly [3, 18, 19, 37, 40, 41, 43, 58–61]. Hence, ROS accumulation and damage to mtDNA might explain why irinotecan attenuated the expression of mitochondrial mRNAs.

This work provides evidence that drugs that evoke DNA replication stress and DNA damage promote metabolic adaptations. Intriguingly, we noted that irinotecan enhanced the protein expression of ETC complex subunits in vivo and in vitro. Chloramphenicol, which is an inhibitor of mitochondrial ribosomes [42], reduced the expression of ETC CI-IV and the irinotecan-induced abundance of ETC proteins and respiration in HCT116 cells. Thus, the higher level of ETC complex subunits during irinotecan-induced DNA replication stress likely originated from increased mitochondrial protein translation, and not from increased mtDNA-encoded mRNA levels or an accumulation of mitochondria. However, further studies will be necessary to consolidate this finding. The mitochondrial DNA harbors TOP1mt, which strictly locates to mitochondria, and they import Top2β and Top3α to solve topological stress [62, 63]. The nuclear topoisomerase TOP1 is not found in mitochondria. Nevertheless, there is evidence that camptothecin (CPT) can inhibit both TOP1 and TOP1mt [64, 65]. This might also apply to the CPT derivate irinotecan (CPT-11) [32, 46, 64] and give an additional explanation for the reduced mtDNA expressions in irinotecan-treated cells.

We validated our biochemical analysis on cell metabolism during DNA replication stress by a targeted metabolomics approach. We found an increased turnover of metabolites from the glycolysis, PPP, and TCA cycle in both HCT116 cell lines. This led to a higher consumption of energy sources from the environmental cell culture medium, increased intracellular metabolite levels, and ATP production. Others have consistently shown that suppression of the cancer cell metabolism, at the levels of mtDNA translation, glucose or glutamine metabolism, can augment the effectiveness of chemotherapy [3, 14, 15, 18, 19, 25–28, 42, 47, 56, 66, 67]. Moreover, in response to mtDNA damage, cancer cells can increase their glutamine metabolism for compensation [19] and metabolic adaptation of the TCA cycle is required for tumor growth in vivo [12]. As expected from these insights, both HCT116 cell lines increase their glutamine metabolism in response to irinotecan. This metabolic adaption can feed the TCA and OXPHOS for energy production, and it is in addition to the PPP, another source for nucleotide and glutathione de novo synthesis [2, 14, 19, 30, 54, 56]. Glutathione is an essential endogenous ROS scavenger and nucleotides are necessary for the repair of DNA damage during chemotherapy [15, 30, 56, 68, 69]. Hence, their production through different metabolic pathways might help CRC cells to deal with DNA replication stress, and an abrogated glucose metabolism appears attractive to increase the effectiveness of chemotherapy.

Interestingly, HCT116^wt^ cells deplete their intracellular glucose and pyruvate levels during DNA replication stress and glucose is a limiting factor for the survival of irinotecan-treated HCT116^wt^ cells. This was also reflected by the OXPHOS activity of HCT116^wt^ cells cultured for 48 h in DMEM with low glucose compared with cells in a high-glucose medium. Isogenic p53-deficient cells use their glucose more economically and were significantly less affected by irinotecan under both high- and low-glucose cell culture conditions. Thus, the high metabolism in HCT116^wt^ cells might protect them from toxic DNA damage if glucose is available, which makes them in turn vulnerable to an absence of this energy source.

## Conclusions

Global analyses illustrate that the irinotecan-induced DNA replication stress/DNA damage leads to metabolic adaptation of CRC cells, including increased OXPHOS, glycolysis, glutaminolysis, TCA, and PPP activity. Hence, exploiting CRC metabolism can be a promising approach to increase the effectiveness of irinotecan and other DNA replication stress-inducing agents in p53-proficient CRC cells.

## Methods

### Reagents

Irinotecan hydrochloride, 2-thenoyltrifluoroacetone (TTFA), hydroxyurea (HU), 2′,7′-dichlorofluorescin diacetate (DCFDA), 3-(4,5-dimethylthiazol-2-yl)-2,5-diphenyltetrazolium bromide (MTT), 2-deoxy-d-glucose (2-DG), crystal violet, and antimycin A were purchased from Sigma Aldrich (Deisenhofen, Germany). Doxorubicin was purchased from Enzo Life Science (Lörrach, Germany). Chloramphenicol was purchased from Carl Roth GmbH (Karlsruhe, Germany). Rotenone, carbonyl cyanide-4-(trifluoromethoxy)phenylhydrazone (FCCP), and oligomycin were purchased from Abcam (Cambridge, UK). 3,3′-Dihexyloxacarbocyanine iodide (DiOC_6_(3)) was purchased from Thermo Fisher Scientific (Waltham, MA, USA). DMSO was used as a treatment control.

### Cell culture

HCT116 wild-type and otherwise isogenic cell lines lacking p53 cells were a gift from Dr. B. Vogelstein (Baltimore, MD, USA). RKO ATCC cells were received from the DSMZ Braunschweig and a gift of Dr. M. Zörnig (Frankfurt/Main, Germany). Cells were maintained in high-glucose (4.5 g/l) DMEM with stable glutamine, 10% fetal calf serum (FCS), and 100 U/ml penicillin/streptomycin (all from Thermo Scientific). Cells were cultivated at 37 °C in a humidified 5% CO_2_ incubator and routinely passaged.

Experiments were performed in high-glucose (4.5 g/l) or low-glucose (1 g/l) DMEM with stable glutamine and 10% fetal calf serum (all from Thermo Scientific) without antibiotics. Cells were treated with 10 µM irinotecan, 1 mM HU, 1 µM doxorubicin, 0.1 mM TTFA, 0.1 µM rotenone, or 0.1 mg/ml chloramphenicol for up to 48 h.

### Flow cytometric analysis of cell death and mitochondrial transmembrane potential (ΔΨ_M_)

Analysis was performed as described in [70].

### Flow cytometric analysis of reactive oxygen species (ROS)

Analysis was performed as described in [70].

### MTT cell viability assays

MTT assays were performed as described in [70]. In parallel, cell numbers were counted using a Particle Counter Z1 (Beckman Coulter) and the obtained MTT values were normalized to corresponding cell numbers.

### Analysis of O_2_ consumption

Detached cells were counted with a hemocytometer (Paul Marienfeld GmbH & Co. KG, Lauda Königshofen, Germany) centrifuged and resuspended to a final concentration of 3 × 10^6^ cells per ml in high-glucose DMEM. One milliliter of this suspension was transferred into a Clarke electrode (Oxygraph system, Hansatech-instruments, Norfolk, UK). O_2_ consumption was measured by the decrease of oxygen within the chamber during a 5-min time period.

### Seahorse analysis of cells

#### Cell Mito Stress Test

The supernatant medium was replaced with a Seahorse XF base medium (Agilent Technologies, pH adjusted to 7.4), supplemented with 10 mM d-glucose (Sigma Aldrich), 2 mM l-glutamine, and/or 1 mM sodium pyruvate (both from Thermo Scientific). Cells were then incubated for additional 1 h in a CO_2_-free incubator at 37 °C. Basal oxygen consumption rate (OCR) and extracellular acidification rate (ECAR) were measured with a Seahorse XFe24 Analyzer (Agilent Technologies) before consecutively adding 2 µM oligomycin, 2 µM FCCP, and 2 µM antimycin A. In this assay, basal OCR and ECAR values are measured before different ETC inhibitors are injected consecutively during the analysis. Oligomycin blocks ETC CV to analyze the amount of OCR used to generate ATP. FCCP chemically disrupts the mitochondrial membrane integrity and membrane potential (ΔΨ_M_) to uncouple the respiration, usually leading to a maximal increase in OCR and ECAR [44, 45]. Antimycin A inhibits ETC CIII (cytochrome *c* reductase) and thus the electron transport from ETC CI/II into CIV. This finally breaks down the mitochondrial respiration [44, 45].

#### Glycolysis Stress Test

The supernatant medium was replaced with a Seahorse XF base medium (pH adjusted to 7.4), supplemented with 2 mM l-glutamine. Cells were then incubated for additional 1 h in a CO_2_-free incubator at 37 °C. Basal OCR and ECAR were measured with a Seahorse XFe24 Analyzer before consecutively adding 10 mM d-glucose, 2 µM oligomycin, and 50 mM 2-deoxy-d-glucose (2-DG). In this assay, d-glucose is added to analyze the resulting increase of ECAR. Oligomycin blocks the OXPHOS and in consequence induces high ECAR in cells to maintain their overall energy production. 2-Deoxy-d-glucose (2-DG), which is a competitive inhibitor of hexokinase and glucose-6-phosphate isomerase [48], breaks down the glycolysis and resulting ECAR.

In both assays, OCR and ECAR were measured as pmol/min and mpH/min, respectively, in cycles of 3-min mix and 3-min measure periods at 37 °C. At the end of the measurement, cell densities per well were quantified by crystal violet staining (performed as described in [70]) and the observed OCR and ECAR were normalized to corresponding cell densities. Wave software (Agilent Technologies) was used to analyze the datasets.

### Quantitative real-time RT–PCR

Total RNA was isolated using the Peqgold Total RNA Kit including DNase digestion (Peqlab, Erlangen, Germany). RNA was transcribed into cDNA using Omniscript (Qiagen, Hilden, Germany). Quantitative PCR for *MT-COII*, *MT-ND1*, and *MT-CYB* was performed using the Applied Biosystems (Darmstadt, Germany) 7900HT Real-Time PCR system. Expression levels were normalized to β2-microglobulin. Reactions were done in duplicate using Applied Biosystems Gene Expression Assays (COX2: Hs02596865_g1, NDH: Hs02596873_s1, CYTB: Hs02596867_s1, β-2-microglobulin: Hs00187842_m1) and Universal PCR Master Mix. All procedures were performed according to the manufacturers’ protocols. The relative gene expressions were calculated by the 2(-^ΔΔ^Ct) method.

### Tumor resection lysates

Resection specimens of human CRC xenografts were obtained from mice as described in [38]. Mice were bred in the animal facility of the University Medical Center in Rostock under specific pathogen-free conditions. During their whole lifetime, all animals got enrichment in the form of mouse-igloos (ANT Tierhaltungsbedarf, Buxtehude, Germany), nesting material (shredded tissue paper, Verbandmittel GmbH, Frankenberg, Germany), paper roles (75 × 38 mm, H 0528–151, ssniff-Spezialdiäten GmbH, Cologne, Germany), and wooden sticks (40 × 16 × 10 mm, Abedd, Vienna, Austria). During the experiment, mice were kept in type III cages (Zoonlab GmbH, Castrop-Rauxel, Germany) at 12-h dark:light cycle, the temperature of 21 ± 2 °C, and relative humidity of 60 ± 20% with food (pellets, 10 mm, ssniff-Spezialdiäten GmbH, Soest, Germany) and tap water ad libitum. Tumor resection specimens were homogenized in radioimmunoprecipitation assay (RIPA) buffer (50 mM Tris/HCl (pH 8.0), 150 mM NaCl, 1 mM EDTA, 1% NP-40, 1% sodium deoxycholate, and 0.1% SDS; supplemented with a protease inhibitor cocktail and 0.5 mM PMSF) followed by rigorous sonication. Protein quantification was done by Pierce BCA Protein Assay Kit (Thermo Scientific) following the manufacturer’s instruction.

### Whole cell lysates

Cell lysates were prepared as described in [70]. Protein quantification was done by Pierce BCA Protein Assay Kit (Thermo Scientific) following the manufacturer’s instruction.

### Mitochondrial isolation

Mitochondria were isolated as described in [71].

### Immunoblotting

Ten to 20 µg of protein per lane was separated by standard SDS–PAGE and transferred onto PVDF membranes. After blocking in blocking solution (BS; 100 mM Tris/HCl (pH 8.0), 450 mM NaCl, 5% dry milk, and 0.05% Tween-20), the membranes were incubated with antibodies against Total OXPHOS Human WB Antibody Cocktail (1:5000 in BS, Abcam), TOM40 (1:5000), p53 (1:5000 in BS, both from Santa Cruz Biotechnology, Dallas, TX, USA), and phospho-Ser139 Histone H2AX (γH2AX) (1:5000 in BS, Millipore/Merck KGaA, Darmstadt, Germany). Equal loading of whole cell lysates was verified by the detection of HSP90 (1:5000 in BS, Santa Cruz). Peroxidase-conjugated anti-mouse IgG (H + L) and anti-rabbit IgG (H + L) secondary antibodies (1:10,000; Abcam) were used and detection of specific signals was done with SuperSignal West Pico Chemiluminescent Substrate (Pierce/Thermo Scientific). Densitometric quantification was done with ImageJ (National Institutes of Health, Bethesda, MD, USA). Full immunoblot images are depicted in supplementary Fig. S4.

### Mass spectrometric analysis (metabolomics)

LC–MS analysis was performed as described in [30, 49]. Metabolites were extracted from supernatant media before and after treatment and by lysing cells in ice-cold methanol/acetonitrile/H_2_O (50:30:20). Samples were shaken at 4 °C for 10 min and then centrifuged for 15 min at 16,000 *g*, and the supernatant was collected and analyzed by LC–MS. Analytes were separated using hydrophilic interaction liquid chromatography with a SeQuant ZIC-pHILIC column (2.1 3 150 mm, 5 mm) (Merck) and detected with high-resolution, accurate-mass mass spectrometry using an Orbitrap Exactive in line with an Accela autosampler and an Accela 600 pump (Thermo Scientific). The elution buffers were acetonitrile for buffer A and 20 mM (NH_4_)_2_CO_3_ and 0.1% NH_4_OH in H_2_O for buffer B. A linear gradient was programmed starting from 80% buffer A and ending at 20% buffer A after 20 min, followed by wash (20% buffer A) and re-equilibration (80% buffer A) steps with a flow rate of 100 ml/min. The mass spectrometer was fitted with an electrospray-ionization probe and operated in full-scan and polar-switching mode with the positive voltage at 4.5 kV and negative voltage at 3.5 kV. Metabolite identification and data analysis were carried out using LCQUAN software (Thermo Scientific).

### Statistical analysis

Statistical analyses were done using two-tailed Student’s *t* test with Microsoft Excel (*p < 0.05, **p < 0.01, ***p < 0.001). All values are displayed as mean ± SEM.

## Supplementary Information

**Additional file 1: Fig. S1.** (A-B) p53 wild-type (HCT^wt^) and isogenic p53 null (HCT^Δp53^) HCT116 cells were exposed to 10 µM irinotecan (Iri). (A) The expression of indicated proteins in whole cell lysates was analyzed after 24 h by Western blot. HSP90 was used to control equal protein loading. (B) The oxygen consumption of cells was assessed at indicated time points after treatment using a Clarke electrode. (C) HROC24 and HROC87 patient-derived short term colorectal cancer cells were xenotransplanted into mice and treated with Iri or control (see methods). The expression of indicated proteins in tissue lysates of tumor resections was analyzed by Western blot. The expression of electron transport chain (ETC) complex subunits relative to complex V was quantified by densitometry using ImageJ and is depicted next to the immunoblots. (D) MTT assays were performed, and corresponding cell numbers counted in parallel to calculate the MTT turnover/cell number (refer to Fig. 1D). HCT^Δp53^ (E) and HCT^wt^ (F) cells were exposed to 10 µM Iri, 1 µM hydroxyurea (HU), 1 µM doxorubicin (Doxo), 0.1 mM TTFA, 0.1 µM rotenone (Rot) or 0.1 mg/ml chloramphenicol (Chlor) as single agents and in combination for 24 h. The oxygen consumption of cells was assessed using a Clarke electrode. (A) is representative of 2 individual experiments. (C) The number of individual experimental repeats is depicted within the graphs/bars representing their average ± SEM. (B, D-F) show the average of 3 individual experiments ± SEM. Statistics for this figure: * *p*<0.05; ** *p*<0.01; *** *p*<0.001.

**Additional file 2: Fig. S2.** (A-B) p53 wild-type (HCT^wt^) and isogenic p53 null (HCT^Δp53^) HCT116 cells were exposed to 10 µM irinotecan (Iri) for 24 h. Oxygen consumption (OCR) and extracellular acidification rates (ECAR) were assessed using a Seahorse XFe24 Analyzer. The workflow of a standard Cell Mito Stress Test is depicted in (A). (B) Basal OCR and ECAR values were calculated from curves shown in Fig. 2A-B. (C-E) p53 wild-type RKO cells were exposed to 10 µM irinotecan (Iri) for 24 h. (C) Oxygen consumption (OCR) and (D) extracellular acidification rates (ECAR) were assessed with a Cell Mito Stress Test using a Seahorse XFe24 Analyzer. 2 µM oligomycin, 2 µM FCCP and 2 µM antimycin A were injected consecutively during the measurement. (E) Bioenergetic parameters, i.e., ATP production and respiratory spare capacity, were calculated from OCR/ECAR curves shown in (C-D). (F-G) HCT^wt^ and HCT^Δp53^ cells were exposed to 10 µM irinotecan (Iri) for 24 h. Oxygen consumption (OCR) and extracellular acidification rates (ECAR) were assessed using a Seahorse XFe24 Analyzer. The workflow of a Glycolysis Stress Test is depicted in (F). (G) The glutamine- and glucose-driven OCR calculated from curves shown in Fig. 2D. Abbreviations: mitochondrial membrane potential (MMP), electron transport chain (ETC), glutamine (gln), glucose (glu) and pyruvate (pyr). (B, G) show the average of 3 individual experiments ± SEM. (C-E) show the average of 2 individual experiments ± SEM. Statistics for this figure: * *p*<0.05; ** *p*<0.01; *** *p*<0.001.

**Additional file 3: Fig. S3. ** p53 wild-type (HCT^wt^) and isogenic p53 null (HCT^Δp53^) HCT116 cells were exposed to 10 µM irinotecan (Iri) for 24 h. The workflow of a custom Cell Mito Stress Test in the absence of pyruvate is depicted in (A). (B) Oxygen consumption (OCR) and (C) extracellular acidification rates (ECAR) were assessed using a Seahorse XFe24 Analyzer. 2 µM oligomycin, 2 µM FCCP and 2 µM antimycin A were injected consecutively during the measurement. (D) Basal OCR and ECAR values were calculated from curves shown in (B-C). (E) Bioenergetic parameters, i.e., ATP production and respiratory spare capacity, were calculated from OCR/ECAR curves shown in (B-C). The workflow of a custom Cell Mito Stress Test in the absence of glucose is depicted in (F). (G) OCR and (H) ECAR were assessed using a Seahorse XFe24 Analyzer. 2 µM oligomycin, 2 µM FCCP and 2 µM antimycin A were injected consecutively during the measurement. (I) Basal OCR and ECAR values were calculated from curves shown in (G-H). (J) Bioenergetic parameters, i.e., ATP production and respiratory spare capacity, were calculated from OCR/ECAR curves shown in (G-H). Abbreviations used: mitochondrial membrane potential (MMP), electron transport chain (ETC), glutamine (gln), glucose (glu) and pyruvate (pyr). The data presented in (B-E) and (G-J) were generated side-by-side, and all values are normalized to control values of HCT^wt^ cells in (B) and (C), respectively. All graphs show the average of 3 individual experiments ± SEM. Statistics for this figure: * *p *< 0.05; ** *p *< 0.01; *** *p *< 0.001 within each panel; ° *p *< 0.05; °° *p *< 0.01; °°° *p *< 0.001 in comparison between (D) and (I) as well as (E) and (J), respectively.

**Additional file 4: Fig. S4.** (A) and (B) show the full immunoblots used to assemble figures 1B and 1H, respectively. (C) and (D) show the full immunoblots used to assemble supplementary figures S1B and S1C, respectively. Black boxes indicate the cropped portion of each immunoblot shown in the corresponding main figures. Immunoblots shown in (D) were detected using a LI-COR Odyssey® detection system. All other figures were detected using ECL and X-ray films.

## Data Availability

Metabolomics datasets used and analyzed during the current study are available from the corresponding author on reasonable request. Otherwise, all data generated or analyzed during this study are included in this published article (and its supporting information files).

## Abbreviations

AMPK: 5′ Adenosine monophosphate (AMP)-activated protein kinase
ATP: Adenosine triphosphate
Chlor: Chloramphenicol
COX: Cytochrome *c* oxidase
CPT: Camptothecin
CRC: Colorectal cancer cells
DCFDA: 2′,7′-Dichlorofluorescin diacetate
ECAR: Extracellular acidification rate
ETC: Electron transport chain
ETC CI-V: ETC complex I-V
FCCP: Carbonyl cyanide-4-(trifluoromethoxy)-phenylhydrazone
GSH/GSSG: Reduced/oxidized glutathione
γH2AX: Phosphorylated histone protein H2AX
HCC: Hepatocellular carcinoma
HCT116^wt^: HCT116 cell line expressing wild-type p53 (wt)
HCT116^Δp53^: HCT116 cell line with p53 deletion (Δp53; p53 null; p53-/-)
HROC: Hansestadt Rostock colon cancer
HU: Hydroxyurea
Iri: Irinotecan (CPT-11)
KRAS: Kirsten rat sarcoma virus
MMP (ΔΨ_M_): Mitochondrial membrane potential
mtDNA: Mitochondrial DNA
mTOR: Mechanistic target of rapamycin
MTT: 3-(4,5-Dimethylthiazol-2-yl)-2,5-diphenyltetrazolium bromide
NDH: NADH dehydrogenase
OCR: Oxygen consumption rate
OXPHOS: Oxidative phosphorylation
PEP: Phosphoenolpyruvate
PGC1α: Peroxisome proliferator-activated receptor gamma coactivator 1-alpha
PPP: Pentose phosphate pathway
qPCR: Quantitative polymerase chain reaction
RNR: Ribonucleotide reductase
ROS: Reactive oxygen species
SDH: Succinate dehydrogenase
SIRT1: Sirtuin 1
TCA: Tricarboxylic acid cycle
TOP1: Topoisomerase-1
TOP1mt: Mitochondrial topoisomerase-1
TOP2: Topoisomerase-2
TTFA: 2-Thenoyltrifluoroacetone
