# Supplementary figures and images for: Global metabolic alterations in colorectal cancer cells during irinotecan-induced DNA replication stress
Source: Cancer Metab. 2022 Jul 4;10:10. doi: 10.1186/s40170-022-00286-9 (PMC9251592; doi:10.1186/s40170-022-00286-9)

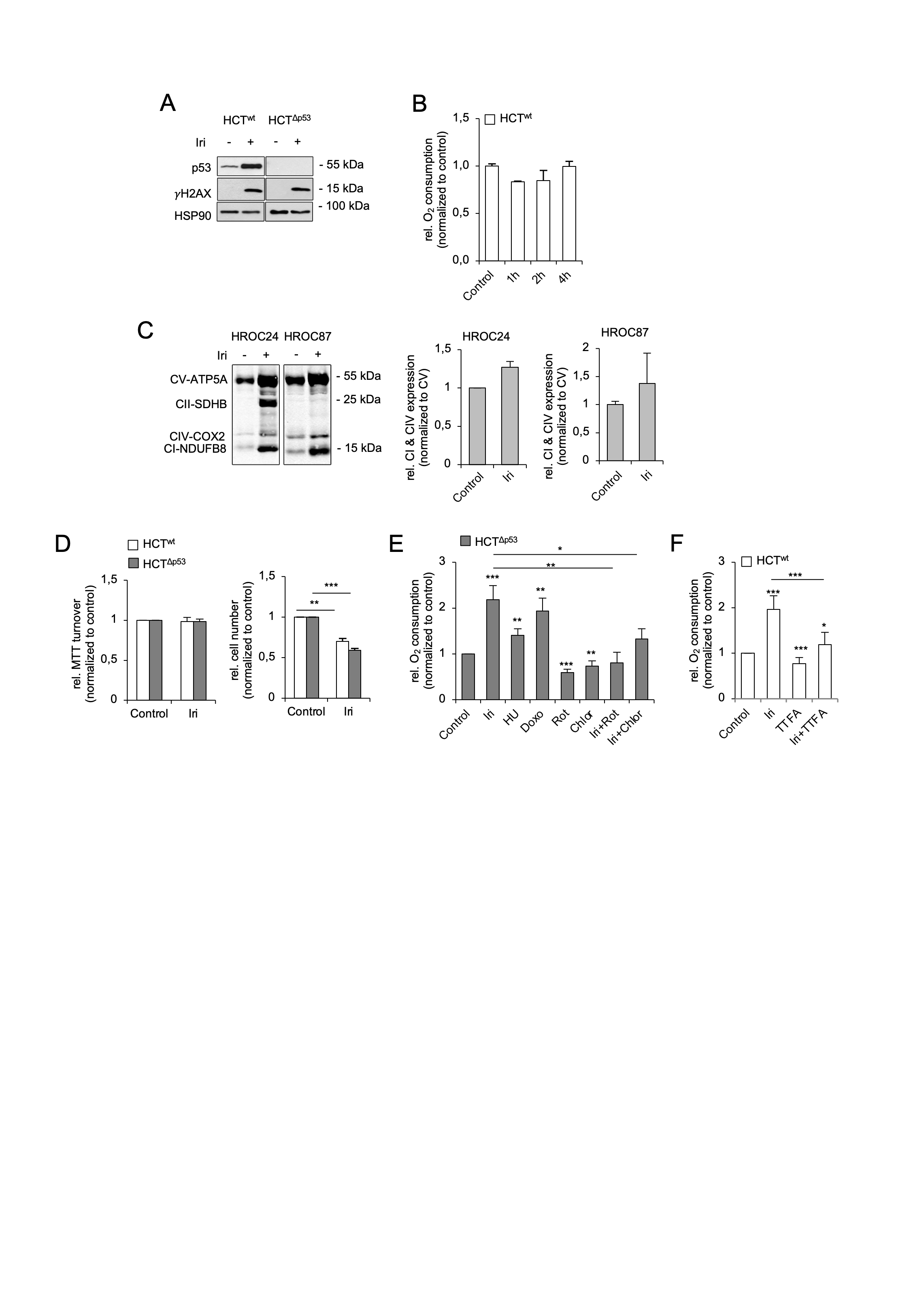

Supplement: Supplementary file 1 — Additional file 1: Fig. S1. (A-B) p53 wild-type (HCTwt) and isogenic p53 null (HCTΔp53) HCT116 cells were exposed to 10 µM irinotecan (Iri). (A) The expression of indicated proteins in whole cell lysates was analyzed after 24 h by Western blot. HSP90 was used to control equal protein loading. (B) The oxygen consumption of cells was assessed at indicated time points after treatment using a Clarke electrode. (C) HROC24 and HROC87 patient-derived short term colorectal cancer cells were xenotransplanted into mice and treated with Iri or control (see methods). The expression of indicated proteins in tissue lysates of tumor resections was analyzed by Western blot. The expression of electron transport chain (ETC) complex subunits relative to complex V was quantified by densitometry using ImageJ and is depicted next to the immunoblots. (D) MTT assays were performed, and corresponding cell numbers counted in parallel to calculate the MTT turnover/cell number (refer to Fig. 1D). HCTΔp53 (E) and HCTwt (F) cells were exposed to 10 µM Iri, 1 µM hydroxyurea (HU), 1 µM doxorubicin (Doxo), 0.1 mM TTFA, 0.1 µM rotenone (Rot) or 0.1 mg/ml chloramphenicol (Chlor) as single agents and in combination for 24 h. The oxygen consumption of cells was assessed using a Clarke electrode. (A) is representative of 2 individual experiments. (C) The number of individual experimental repeats is depicted within the graphs/bars representing their average ± SEM. (B, D-F) show the average of 3 individual experiments ± SEM. Statistics for this figure: * p<0.05; ** p<0.01; *** p<0.001. [file 40170_2022_286_MOESM1_ESM.tiff]

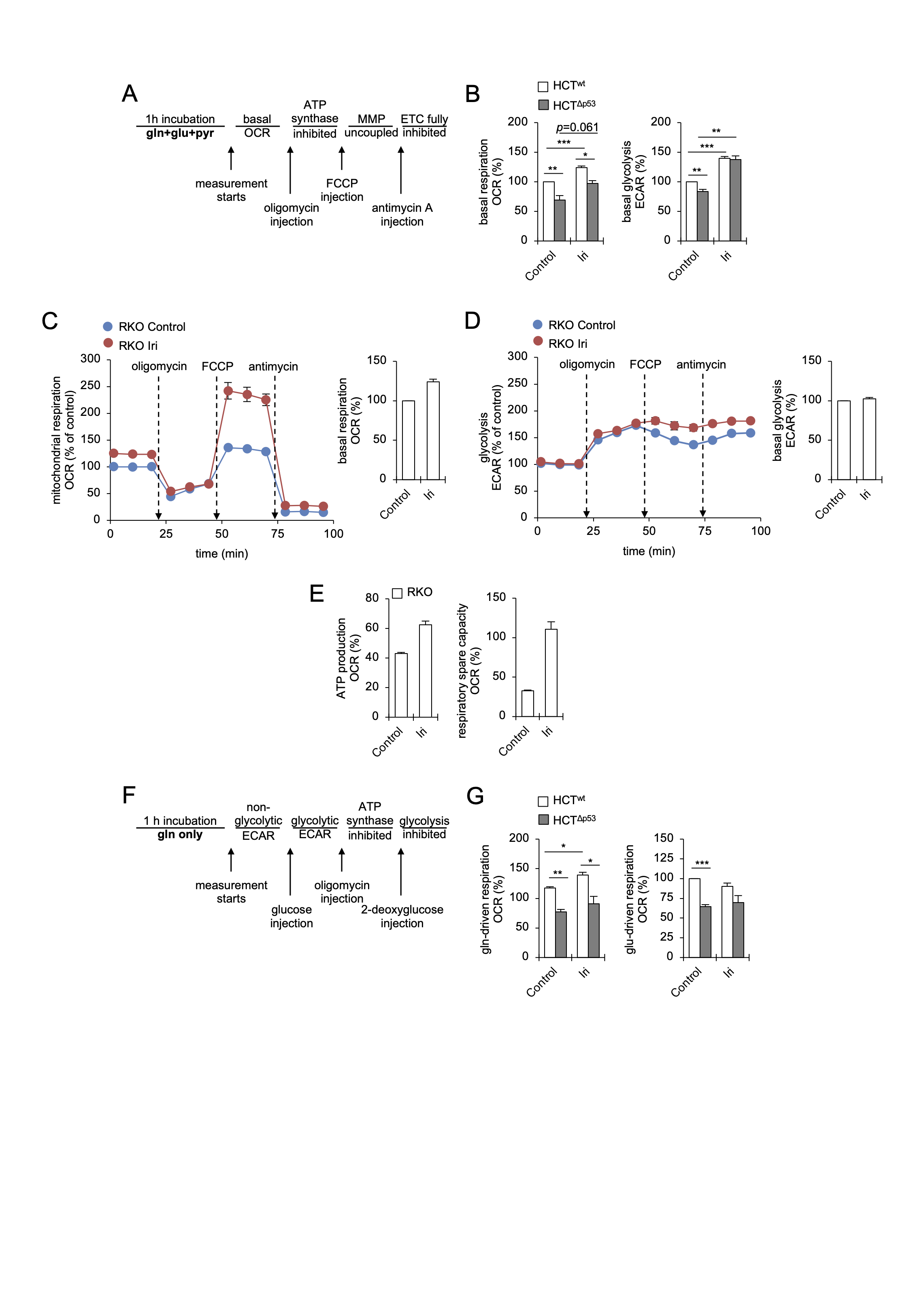

Supplement: Supplementary file 2 — Additional file 2: Fig. S2. (A-B) p53 wild-type (HCTwt) and isogenic p53 null (HCTΔp53) HCT116 cells were exposed to 10 µM irinotecan (Iri) for 24 h. Oxygen consumption (OCR) and extracellular acidification rates (ECAR) were assessed using a Seahorse XFe24 Analyzer. The workflow of a standard Cell Mito Stress Test is depicted in (A). (B) Basal OCR and ECAR values were calculated from curves shown in Fig. 2A-B. (C-E) p53 wild-type RKO cells were exposed to 10 µM irinotecan (Iri) for 24 h. (C) Oxygen consumption (OCR) and (D) extracellular acidification rates (ECAR) were assessed with a Cell Mito Stress Test using a Seahorse XFe24 Analyzer. 2 µM oligomycin, 2 µM FCCP and 2 µM antimycin A were injected consecutively during the measurement. (E) Bioenergetic parameters, i.e., ATP production and respiratory spare capacity, were calculated from OCR/ECAR curves shown in (C-D). (F-G) HCTwt and HCTΔp53 cells were exposed to 10 µM irinotecan (Iri) for 24 h. Oxygen consumption (OCR) and extracellular acidification rates (ECAR) were assessed using a Seahorse XFe24 Analyzer. The workflow of a Glycolysis Stress Test is depicted in (F). (G) The glutamine- and glucose-driven OCR calculated from curves shown in Fig. 2D. Abbreviations: mitochondrial membrane potential (MMP), electron transport chain (ETC), glutamine (gln), glucose (glu) and pyruvate (pyr). (B, G) show the average of 3 individual experiments ± SEM. (C-E) show the average of 2 individual experiments ± SEM. Statistics for this figure: * p<0.05; ** p<0.01; *** p<0.001. [file 40170_2022_286_MOESM2_ESM.tiff]

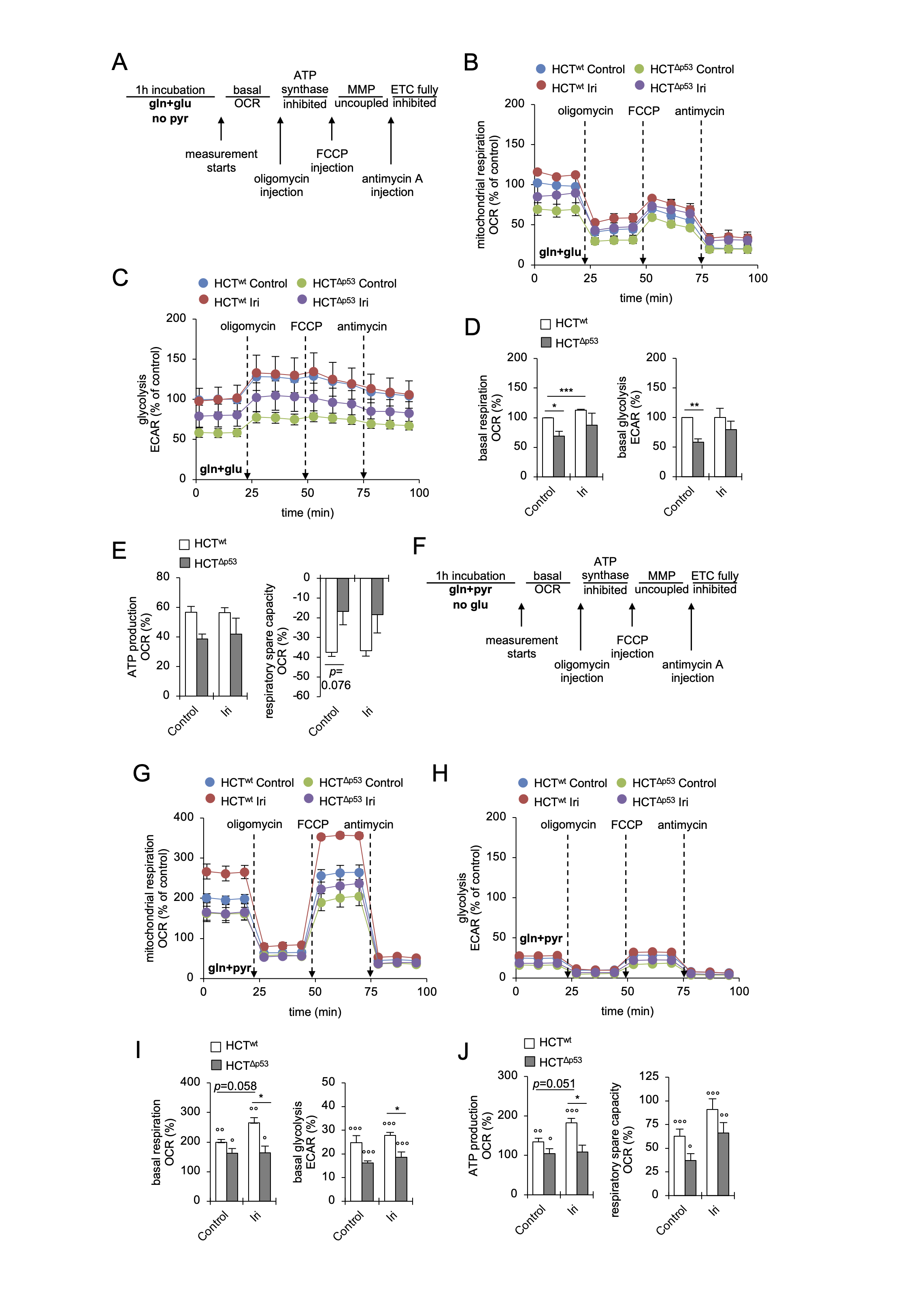

Supplement: Supplementary file 3 — Additional file 3: Fig. S3. p53 wild-type (HCTwt) and isogenic p53 null (HCTΔp53) HCT116 cells were exposed to 10 µM irinotecan (Iri) for 24 h. The workflow of a custom Cell Mito Stress Test in the absence of pyruvate is depicted in (A). (B) Oxygen consumption (OCR) and (C) extracellular acidification rates (ECAR) were assessed using a Seahorse XFe24 Analyzer. 2 µM oligomycin, 2 µM FCCP and 2 µM antimycin A were injected consecutively during the measurement. (D) Basal OCR and ECAR values were calculated from curves shown in (B-C). (E) Bioenergetic parameters, i.e., ATP production and respiratory spare capacity, were calculated from OCR/ECAR curves shown in (B-C). The workflow of a custom Cell Mito Stress Test in the absence of glucose is depicted in (F). (G) OCR and (H) ECAR were assessed using a Seahorse XFe24 Analyzer. 2 µM oligomycin, 2 µM FCCP and 2 µM antimycin A were injected consecutively during the measurement. (I) Basal OCR and ECAR values were calculated from curves shown in (G-H). (J) Bioenergetic parameters, i.e., ATP production and respiratory spare capacity, were calculated from OCR/ECAR curves shown in (G-H). Abbreviations used: mitochondrial membrane potential (MMP), electron transport chain (ETC), glutamine (gln), glucose (glu) and pyruvate (pyr). The data presented in (B-E) and (G-J) were generated side-by-side, and all values are normalized to control values of HCTwt cells in (B) and (C), respectively. All graphs show the average of 3 individual experiments ± SEM. Statistics for this figure: * p < 0.05; ** p < 0.01; *** p < 0.001 within each panel; ° p < 0.05; °° p < 0.01; °°° p < 0.001 in comparison between (D) and (I) as well as (E) and (J), respectively. [file 40170_2022_286_MOESM3_ESM.tiff]

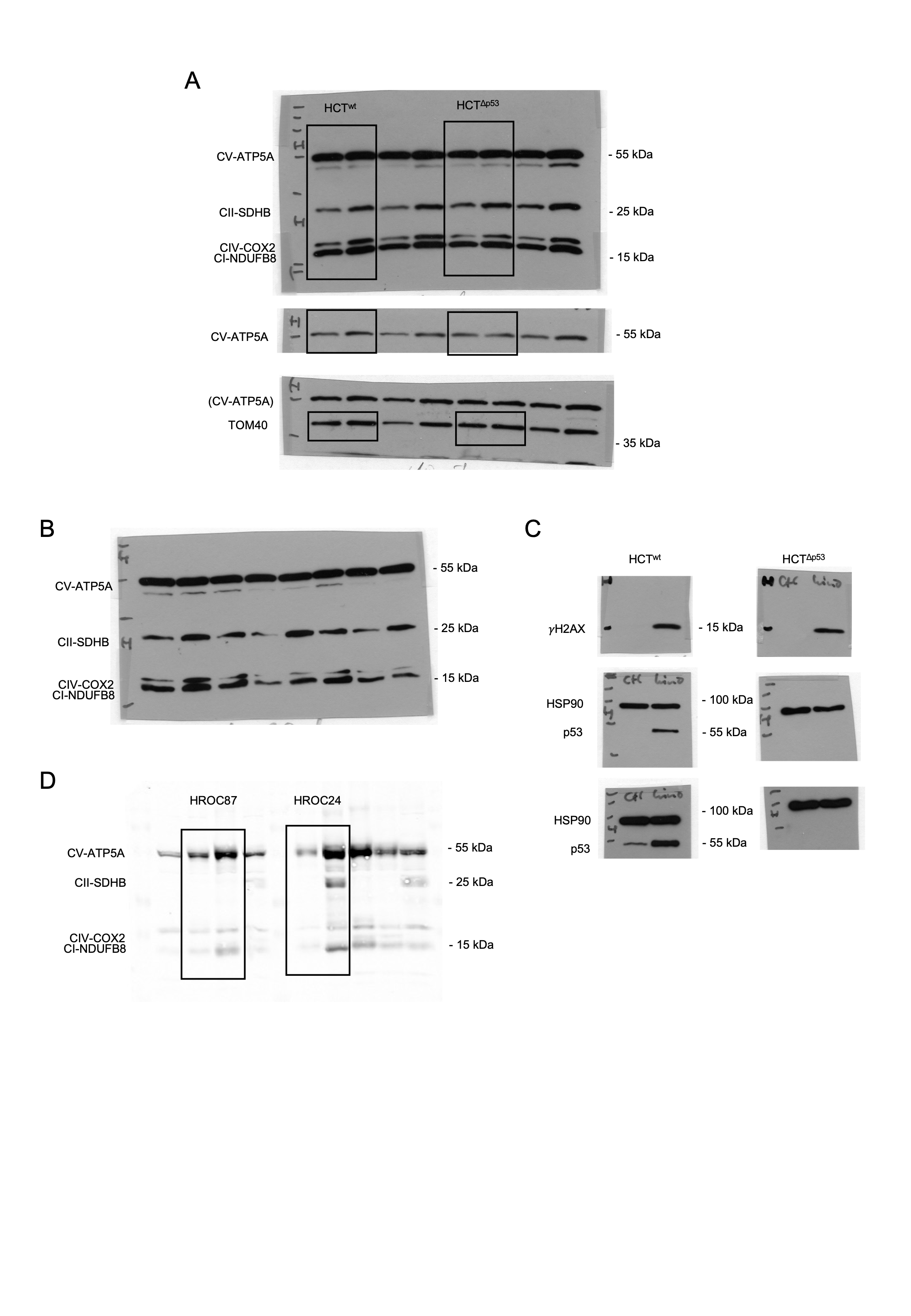

Supplement: Supplementary file 4 — Additional file 4: Fig. S4. (A) and (B) show the full immunoblots used to assemble figures 1B and 1H, respectively. (C) and (D) show the full immunoblots used to assemble supplementary figures S1B and S1C, respectively. Black boxes indicate the cropped portion of each immunoblot shown in the corresponding main figures. Immunoblots shown in (D) were detected using a LI-COR Odyssey® detection system. All other figures were detected using ECL and X-ray films. [file 40170_2022_286_MOESM4_ESM.tiff]
